# Fel d1 Blocking Antibodies: A Novel Method to Reduce IgE-Mediated Allergy to Cats

**DOI:** 10.1155/2021/5545173

**Published:** 2021-06-19

**Authors:** Ebenezer Satyaraj, Peichuan Sun, Scott Sherrill

**Affiliations:** Nestlé Purina Research, One Checkerboard Square, Saint Louis, MO 63164, USA

## Abstract

Fel d1 is an important allergen produced by cats that causes IgE reactions in up to 95% of cat-allergic adults. Immunotherapy to reduce human allergy to cats has demonstrated that people have the capacity to produce allergen-specific neutralizing antibodies that block IgE-mediated allergic responses. We wished to determine if “blocking” antibodies could be used to reduce the IgE binding ability of cat allergens prior to their exposure to humans. Here, we describe the characterization of Fel d1-specific antibodies. We demonstrated the efficacy of a rabbit polyclonal and an allergen-specific chicken IgY to bind to Fel d1 in cat saliva and block Fel d1-IgE binding and IgE-mediated basophil degranulation. Fel d1 blocking antibodies offer a new and exciting approach to the neutralization of cat allergens.

## 1. Introduction

Human sensitivity to allergens released by cats is common [1–3]. Cats produce several proteins including Fel d1-Fel d8, haptoglobin, and S100A12 that bind to IgE in cat-allergic individuals [4]. Fel d1 was identified as a major cat allergen in the early 1970s [5]. It is seen as the most potent of the known cat allergens, eliciting IgE responses in >90% of cat-allergic individuals [6]. Produced by sebaceous, salivary, and lacrimal glands of the cat, the highest Fel d1 levels are found in saliva. Fel d1 is transferred from saliva to their hair when cats groom themselves. Cat dander containing Fel d1 allergen is then spread to the environment as small airborne particles [6–10].

Crosslinking of IgE to receptors on mast cell and basophil surfaces causes rapid cellular degranulation and release of chemical mediators that are responsible for clinical symptoms of allergies. Therapies against IgE-mediated allergy include (1) avoidance of the instigating allergen, (2) symptomatic therapies such as antihistamines, steroids, and bronchodilators, and (3) allergen-specific immunotherapy (SIT). All these options have downsides. It is very difficult to achieve 100% avoidance especially given the ubiquitous nature of allergens such as Fel d1 [11]. Symptomatic therapy necessitates ongoing drug administration with potential issues around safety, compliance, and cost. SIT involves repeated administration of increasing doses of allergens to sensitized individuals to produce a diminution of future allergic responses [12]. Despite evidence of clinical success, SIT trials are also littered with reports of lack of clinical efficacy and by safety issues such as adverse allergic responses including, although rarely, anaphylactic shock [13].

Given the limitations of current allergy reduction strategies, we wanted to investigate a novel approach to neutralizing cat allergens. It has been reported that patients receiving SIT therapy developed allergen-specific IgG_4_ “blocking antibodies” that could interact with the allergen, thereby inhibiting its ability to bind to IgE [14–16]. To date, it has not been determined if such “blocking antibodies” would be applicable to reduce the IgE binding ability of allergen at the source, in this case Fel d1 in cat saliva, hair, and dander after the protein has been produced by the cat. We therefore hypothesized that Fel d1 blocking antibodies could reduce immunologically active Fel d1 in cat saliva, hair, and dander and prevent binding to IgE thus blocking the associated allergic mechanisms. To examine this hypothesis, we measured the effects of blocking antibodies against the Fel d1 protein using two approaches: firstly a modified antigen-IgE-chimeric ELISA [17] and then a degranulation assay using a humanized basophil cell line [18].

Fel d1 is a tetramer composed of two noncovalently linked heterodimers [19, 20]. Each 18 kDa heterodimer is composed of two covalently linked polypeptide chains (chains 1 and 2) which lie antiparallel to each other [21]. At least three IgE-specific epitopes have been identified in Fel d1: amino acids 25-38 and 46-59 on chain 1 and amino acids 15-28 on chain 2 [22]. This work and those by others have demonstrated Fel d1-to-IgE binding to be conformational [21]. Multiple IgE binding epitopes are required for allergen-induced crosslinking of mast cell- and basophil-bound IgEs and cellular degranulation [23]. The conformational binding of Fel d1 indicated that a polyclonal antibody targeting multiple epitopes could have the best blocking potential, so this was pursued.

## 2. Materials and Methods

### 2.1. Allergens, Human Plasma, and Cat Saliva Samples

The purified cat major allergen protein Fel d1 and polyclonal antibodies against purified native Fel d1 made in rabbit serum were obtained from Indoor Biotech (VA, USA). The monoclonal rabbit anti-Fel d1 antibody FGI was obtained from FabGennix, Inc. (TX, USA). The chicken egg anti-Fel d1 IgY antibody was harvested from egg yolks from hens inoculated with purified Fel d1. Human plasma samples from allergy-free patients or those with known allergies were purchased from Plasma Lab International (WA, USA). Cat saliva was collected from healthy cats at the Nestlé Purina Petcare Center (Missouri, USA) using a commercially available Salivette® (Sarstedt, Germany). The cats were allowed to chew on the Salivette for about 10-15 seconds, and then, the Salivettes were centrifuged (1000 g for two minutes) at room temperature to obtain the saliva. Following centrifugation, samples were transferred to microtubes and frozen immediately at −80°C.

### 2.2. Allergen Blocking Measured via Fel d1 Direct and Fel d1-Human Plasma Chimeric ELISA

#### 2.2.1. Fel d1 Direct ELISA

Fel d1 concentrations in saliva were determined using a commercial sandwiched ELISA kit from Indoor Biotech (VA, USA), using the manufacturer's directions. Briefly, a monoclonal Fel d1 Ab (clone# 6F9 A4 H1) was coated onto 96-well plates and maintained at 4°C overnight; then, the coated wells were blocked with 1% bovine serum albumin (BSA) in phosphate-buffered saline with Tween-20 (PBS-T) for 30 minutes at room temperature. Diluted Fel d1 control and saliva samples were added to individual wells and incubated for 1 hour at room temperature. After washing three times with PBS-T, 100 *μ*l biotinylated anti-Fel d1 monoclonal Ab (clone# 3E4 C4 C10) was added and incubated for 1 hour at room temperature. Wells were washed again three times with PBS-T, and 100 *μ*l diluted streptavidin-peroxidase (Sigma S5512, 0.25 mg reconstituted in distilled water) was added before incubation for 30 minutes at room temperature. After washing wells, three additional times with PBS-T, 100 *μ*l 1 mM ABTS in 70 mM citrate phosphate buffer (pH 4.2 and 1/1000 dilution of H_2_O_2_) was added for color development and read at 405 nm.

#### 2.2.2. Fel d1-Human Plasma Chimeric ELISA

This ELISA is modified from a model described by others [24]. Briefly, monoclonal Fel d1 Abs (clone# 6F9 A4 H1) diluted to 1/1000 in carbonate buffer was coated on the bottoms of 96-well ELISA plates and kept overnight at 4°C. Cat saliva samples were mixed with blocking reagents, and positive and negative controls were diluted in PBS and preincubated overnight at 4°C. Fel d1 Abs-coated plates were washed three times with PBS-T, and then, the blocking agent 1% BSA in PBS-T was added and incubated for 30 minutes; then, the plates were washed again three times with PBS-T. The preincubated saliva samples were added and plates incubated for 1 hour and then washed five times with PBS-TT. Finally, allergic patient plasma (as a source of Fel d1-specific IgE) was added in a 1 : 5 dilution with 1% BSA-PBS-T and incubated for 90 minutes at room temperature. Plates were washed five times with PBS-T to wash away any unbound IgE, leaving only Fel d1-bound IgE. Diluted biotinylated goat anti-IgE (Fisher #16-10-04, 1 : 4000 in 1% BSA-PBS-T) was added, and the plates were incubated for 1 hour at room temperature; then, streptavidin-peroxide solution (1/1000 dilution in 1% BSA-PBS-T) and its substrate 3,3′,5,5′-tetramethylbenzidine (TMB) were added sequentially to develop the response. Sulfuric acid (0.1 M) was added to stop the reaction, and the plate was read at 450 nm using a standard ELISA plate reader.

### 2.3. Allergenic Responses Measured in Humanized Rat Basophilic Leukemia Cells

The humanized rat basophilic leukemia (RBL) cell line used in this study was kindly provided from Dr. Vogel's laboratory (Paul-Ehrlich Institute, Langen, Germany). In his lab, RBL-2H3 cells (ATCC, Germany) were transfected with cDNA coding for the human high affinity IgE receptor (FcɛR1) chains. The surface expression of the IgE binding alpha-chain was detected by fluorescence-activated cell sorting, and the functional integration of the humanized receptors into the signal transduction cascade was addressed by intracellular calcium mobilization. Mediator release was measured in response to human IgE and a variety of crosslinking allergen preparations. Several clones were obtained that were able to bind to allergen-specific human IgE, and clone RBL-703/9 was used in this study.

Buffers for the *β*-hexosaminidase release assay included Tyrode's buffer (20x stock) made with 0.1370 M NaCl, 0.0027 M KCl, 0.0004 M NaH_2_PO_4_, and 0.0005 M MgCl_2_ × 6H_2_O in 1 l double distilled water, used fresh or stored at 4°C. The 1x diluted Tyrode's buffers were made by diluting Tyrode's buffer 20x stock solution 1 : 20 in double-distilled water with or without 50% D_2_O (Sigma-Aldrich, USA). Release buffer was made to contain 1.4 mM CaCl_2_ × 2H_2_O, 100 mM HEPES, 5.6 mM glucose, and 0.1% BSA in 1x diluted Tyrode's buffer with D_2_O, and pH was adjusted to 7.45 (stable only 2 days when stored at 4°C). Total release buffer was made with 1% Triton X-100 (Sigma-Aldrich, MO) in phosphate-buffered saline. Release stop solution was made with 0.2 M glycine in double-distilled water, adjusted to pH 10.7, and stored at -20°C. Substrate solution was made by dissolving 1.3 mg/ml p-nitrophenyl-*N*-acetyl-*β*-D-glucosamine (Sigma, MO, USA) in 0.1 M Na_2_HPO_4_, then adjusted to pH 4.5 with 0.4 M citric acid, and stored at -20°C.

Polyclonal antibodies against purified native Fel d1 were obtained from Indoor Biotech (VA, USA), and the chicken egg anti-Fel d1 IgY antibody (harvested from egg yolks obtained from hens inoculated with purified Fel d1) were diluted in 1x PBS (1 : 20 to 1 : 20,000). Cat saliva as a source of Fel d1 was added and mixed well and then incubated at room temperature for at least 1 h or overnight at 4 degrees before being used in chimeric ELISA or *β*-hexosaminidase release assay to detect the degree of reduction. The allergen stock solution was prepared from saliva from the same cat preincubated with buffer PBS only.

For the hexosaminidase release assay, RBL-703/9 cells were cultured in Minimum Essential Medium (MEM) supplemented with 5% fetal calf serum (FCS) and 5% glutamine (all from Invitrogen Corporation, CA, USA) for 2 weeks before being used. Purified polyclonal human myeloma IgE was obtained from Biodesign International (ME, USA) and used to sensitize the RBL cells. Goat anti-human Fc-specific IgE Ab (Accurate Chemical and Scientific Corp., NY, USA) was used as the positive control.

The assay was conducted as previously described [18] with minor modification. Briefly, prepared RBL cells were harvested, washed twice with culture media, resuspended, and cell counted to determine the concentration. Then, cells were diluted to 1 × 10^6^ cells/ml in cell culture media and aliquoted to 50 *μ*l/wells in a sterile cell culture-grade 96-well plate. Purified human IgE solutions were added to all wells except wells designed as “no-stimulate” control, and plates were then incubated overnight in a cell culture incubator (37°C, 5% CO_2_). Incubated plates were washed three times with 1x Tyrode's buffer without D_2_O. Total release buffer (100 *μ*l 1% Triton X-100) was added to control wells designated for total release, and 100 *μ*l Tyrode's buffer without D_2_O was added to wells designated as spontaneous release wells.

For the test wells, 100 *μ*l of anti-IgE or allergen dilutions were added, and the plate was incubated for 1 hour in an incubator (37°C, 5% CO_2_). After the incubation, culture supernatants were harvested. In a fresh microtiter plate, 30 *μ*l supernatant was added to 50 *μ*l substrate solution and incubated for 60 min at 37°C. Finally, 100 *μ*l stop solution was added and extinction (OD) determined at 405 nm (reference filter at 620 nm) in an ELISA reader.

Relative release was calculated according to the following formula:

(1) $$\begin{array}{c} \frac{\text{sample}–\text{spontaneous}*100}{\text{total}}. \end{array}$$

### 2.4. Statistical Methods

The Shapiro-Wilk test was used to evaluate normal distribution of data. Subsequently, a one-way analysis of variance (ANOVA) was applied to all data, followed by Dunnett's test when overall treatment differences were significant. A *p* value of <0.05 was considered significant. Finally, Cohen's *D* test was used to determine the size of treatment effects. A value of 0.5 on Cohen's *D* test indicates a medium effect while a value of 0.8 or greater indicates a large effect.

## 3. Results

**Figure 1:**
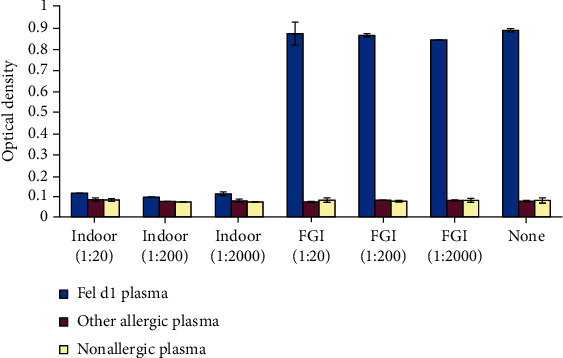
Chimeric ELISA evaluation of a rabbit polyclonal anti-Fel d1 antibody (indoor) and rabbit monoclonal anti-Fel d1 (FGI) on IgE binding in different plasma samples. Chimeric ELISA analysis of IgE blocking effect of rabbit anti-Fel d1 polyclonal “Indoor Poly” (Indoor Biotechnologies, Cat# PA-FD1) and rabbit anti-Fel d1 monospecific “FGI” antibodies (FabGennix International, Cat# FELD1-121AP). Both antibodies were tested at serial dilutions of 1 : 20 to 1 : 2,000,000. Cat saliva samples incubated with buffer only (none) showed high Fel d1-to-IgE binding in cat allergic plasma, but not in noncat allergic plasma and nonallergic plasma. Binding of Fel d1 in cat allergic human plasma was reduced by Indoor Poly versus control (none) at dilutions to 1 : 2000 (*p* < 0.001) but was not affected by the FGI antibody (*p* > 0.10). With noncat allergic and nonallergic plasma, Fel d1 capture was unaffected by all antibodies. ^∗^Significantly different from control (*p* < 0.001).

We selected two commercially available rabbit anti-Fel d1 antibodies for initial assessment: Indoor Poly (Indoor Technologies Inc., Cat# PA-FD1) and “FGI” antibodies (FabGennix International, Cat# FELD1-121AP). Indoor Poly is an antiserum containing rabbit IgG antibodies to multiple Fel d1 epitopes. “FGI” is a monospecific rabbit anti-Fel d1 antibody raised against a peptide sequence covering amino acids 23-40 in chain 1, which is a known IgE binding site [22]. Human plasma samples from cat-allergic, allergic but not to cats, or nonallergic donors provided the source of human IgE. Cat saliva was preincubated with Indoor Poly or FGI antibodies, or “no-antibody” control, and then applied to an ELISA plate coated with a capture antibody (anti-Fel d1 monoclonal clone 6F9). In the “no-antibody” control, the captured Fel d1 bound with high specificity to the anti-Fel d1 IgE was contained in the cat-allergic plasma, with minimal binding to IgE from noncat allergic and control plasma (Figure 1). Preincubation of the Indoor Poly antibody with cat saliva significantly reduced Fel d1 binding to IgE in plasma from cat-allergic subjects at dilutions of 1 : 20 to 1 : 2000 (*p* < 0.001), but no inhibition was noted with the FGI antibody (Figure 1). Cohen's *D* was greater than 90 for dilutions of the Indoor Poly antibody up to 1 : 2000, indicating a large effect when exposed to cat-allergic plasma. The effect of Indoor Poly was dose dependent, being minimal at dilutions of 1 : 200,000 and 1 : 2,000,000 (data not shown).

**Figure 2:**
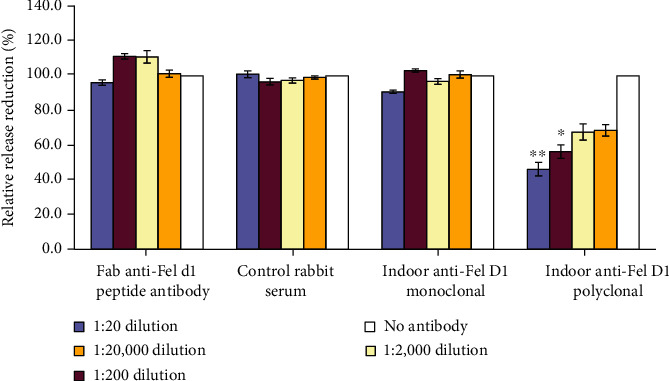
Beta-hexosaminidase release assay with multiple rabbit Fel d1 antibodies and control serum. “Indoor Poly” dose-dependently reduced relative mediator release in the rat basophil assay compared to a “no-antibody” control. Control rabbit serum, FGI, and Fel d1-specific monoclonal (clone 6F9) had no effect on relative mediator release. ^∗^Significantly different from control (*p* < 0.001). ^∗∗^Significantly different from control (*p* < 0.05).

To investigate if the blocking action of Indoor Poly would reduce IgE-induced allergic responses, we probed its action in a rat basophilic leukemia (RBL) cell line stably transfected with human Fc*ε*R1. The binding of antigen-specific IgE to Fc*ε*R1 sensitizes effector cells to release mediators in response to subsequent exposure to that specific antigen. This humanized basophil cell line was previously used to test allergen potency [18]. RBL cells were sensitized by overnight incubation with human plasma from cat-allergic donors. Cell degranulation and mediator release were induced by the addition of cognate allergen. In this case, Fc*ε*R1 crosslinking in the presence of cat saliva containing Fel d1 induced RBL cell degranulation which was measured by quantification of *β*-hexosaminidase release (mediator release). Cat saliva was preincubated with increasing dilutions of the Indoor Poly antibody, FGI antibody, control rabbit serum, or monoclonal anti-Fel d1 (clone 6F9). Results were expressed as a percentage reduction in maximum release measured in a Triton X-100 control in which all cells are lysed by the detergent. Mediator release was dose-dependently blocked (at dilutions to 1 : 200, *p* < 0.05) in saliva samples preincubated with Indoor Poly, but not with the FGI anti-Fel d1 antibody or control rabbit serum (Figure 2). Monoclonal anti-Fel d1 (clone 6F9) also had no blocking action (Figure 2). The effectiveness of Indoor Poly compared to FGI supported our hypothesis that maximal blocking activity is achieved by a polyclonal antibody recognizing multiple epitopes on Fel d1 protein.

**Figure 3:**
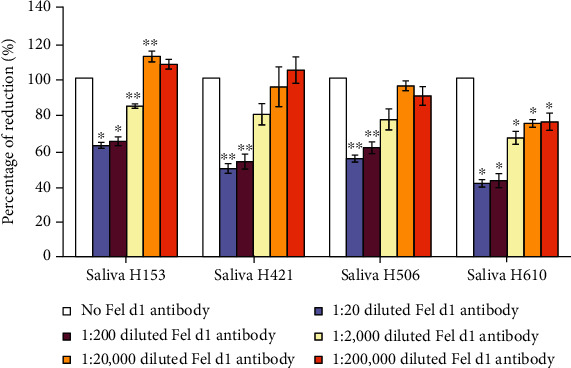
Beta-hexosaminidase release assay with multiple cat saliva samples. The Indoor Poly antibody dose-dependently reduced Fel d1-to-IgE binding and mediator release in multiple cat saliva samples. Although responses differed across samples, there was a dose-dependent decrease in mediator release across saliva samples at dilutions to 1 : 200 (*p* < 0.05). ^∗^Significantly different from control (*p* < 0.001). ^∗∗^Significantly different from control (*p* < 0.05).

Purified Fel d1 has been shown to induce dose-dependent IgE-mediated histamine release in blood samples from cat-allergic individuals [25]. Fel d1 levels can vary in cat saliva; therefore, we repeated the RBL assay using saliva from multiple cats. As before, the Indoor Poly antibody dose-dependently inhibited mediator release, at dilutions to 1 : 2000 (*p* < 0.001) (Figure 3).

**Figure 4:**
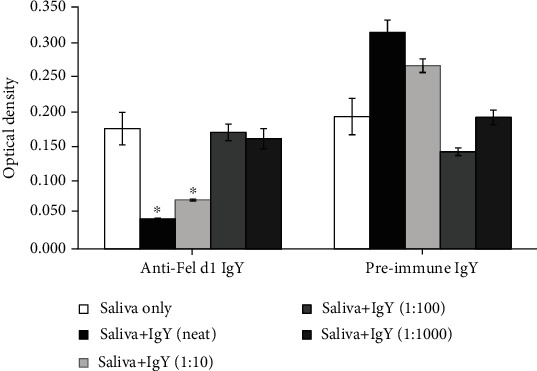
Chimeric ELISA evaluation of chicken egg anti-Fel d1 IgY antibodies on IgE binding compared to egg IgY antibodies from nonimmunized chickens. Anti-Fel d1 IgY antibodies dose-dependently inhibited binding to Fel d1-specific IgE (^∗^*p* < 0.001) while preimmune IgY had no inhibitory action. ^∗^Significantly different from control (*p* < 0.001).

**Figure 5:**
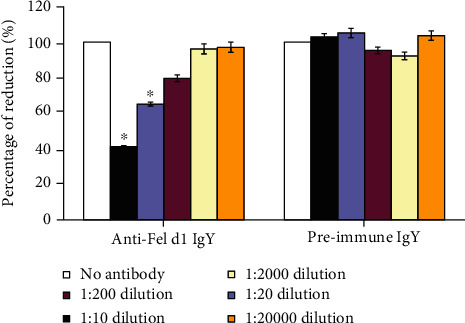
Beta-hexosaminidase release assay with chicken egg anti-Fel d1 antibodies compared to egg IgY antibodies from nonimmunized chickens. The anti-Fel d1 IgY antibody dose-dependently reduced mediator release in the rat basophil assay compared to a “no-antibody” control while preimmune IgY had no effect. ^∗^Significantly different from control (*p* < 0.001).

Chicken egg yolk-derived immunoglobulin Y (IgY) is an alternative high-yield approach to generate antibodies. Multiple studies have proven the efficacy and safety of oral administration of IgY in reducing diarrhea in domesticated animals [26]. We generated Fel d1-specific IgY antibodies and repeated the experiments described above. Similar to Indoor Poly, Fel d1-specific polyclonal IgY antibodies dose-dependently blocked the binding of Fel d1 from cat saliva to Fel d1-specific IgE in our chimeric ELISA (Figure 4), while serum collected preimmunization was without effect. In the humanized basophil assay, anti-Fel d1 IgY blocked mediator release in a dose-dependent manner at dilutions to 1 : 200, thereby demonstrating its physiological relevance (Figure 5).

## 4. Discussion

Here, we report the characterization of antibodies: the first is a commercially available Fel d1-specific polyclonal antibody (Indoor Poly) and the second is partially purified egg yolk-derived polyclonal IgY antibodies raised against Fel d1, which could effectively reduce cat saliva-derived Fel d1-to-human IgE binding and IgE-mediated basophil degranulation. The results presented provide evidence of a novel strategy to reduce IgE accessible Fel d1 levels in cat saliva. These blocking antibodies offer a new and novel approach to the reduction of cat allergens. Fel d1 is relatively abundant in many indoor environments and can be transported on clothing to environments in which no cats are present [11]; therefore, these blocking antibodies could reduce exposure to immunologically active Fel d1. This approach is analogous to the avoidance approach, reducing the likelihood of a sensitized individual encountering the trigger allergen. Egg yolk IgY antibodies are proven to be safe and effective in both companion animals [26] and humans [27], and the blocking activity of the partially purified IgY antibodies was comparable to that of the commercial rabbit polyclonal antibody. Previous studies have shown that the affinity of IgY antibodies is comparable to that of rabbit IgG [28].

The failure of the monospecific polyclonal antibody (FGI) to block IgE binding as compared to the polyclonal Indoor Poly gives insights into the mechanism of action of the effective blocking antibodies. At least three IgE-specific binding sites have been identified on Fel d1, and binding is conformational, requiring correct orientation of chains 1 and 2 for maximal IgE response [20, 21, 28–31]. The FGI antibody recognizes a single epitope among amino acids 23-40, a known IgE binding site on Fel d1 chain 1 [22]. However, additional IgE binding sites between amino acids 46-59 on chain 1 and amino acids 15-28 on chain 2 have been described [31]. Thus, Indoor Poly and our egg yolk IgY likely are more effective through targeting of multiple epitopes on Fel d1 chains 1 and 2. The binding of these multiepitope antibodies to Fel d1 appears to compete with IgE for binding, likely sequestering and neutralizing the allergen as has been demonstrated for human blocking antibodies [14]. A previous study demonstrated that monoclonal antibodies had limited IgE blocking potential; however, a combination of multiple monoclonal antibodies can significantly inhibit IgE binding, an effect the authors contributed to steric hindrance [30]. This and our current findings indicate that maximum blocking can be achieved with multiple epitope antibodies.

## 5. Conclusion

This study provided important findings about blocking antibodies to Fel d1 and demonstrated the potential of a new approach for reducing the allergenicity of Fel d1; nevertheless, the *in vitro* nature of these studies is acknowledged. These *in vitro* studies reported here must be followed with appropriate *in vivo* studies in cats and studies in cat-allergic people to determine if clinically meaningful benefits can be provided. Polyclonal antibodies, such as egg yolk-derived IgY that is known to be well tolerated and effective, offer the potential of a safe and noninvasive way to reduce the allergenicity of cats.

## Data Availability

All data is available in the manuscript.
